# Low-vacuum SEM imaging and viability test of L929 cells exposed to a Euro 6 diesel exhaust gas mixture in a BAT-CELL chamber in comparison with hydrocarbons emission

**DOI:** 10.1038/s41598-024-63560-4

**Published:** 2024-06-05

**Authors:** Aleksandra Kęska, Agnieszka Rusak, Radosław Włostowski, Mikołaj Dziemieszkiewicz, Natalia Szymlet

**Affiliations:** 1 Department of Automotive Engineering, Wroclaw University of Science and Technology, Wybrzeże Wyspiańskiego 27, 50-370 Wrocław, Poland; 2 Department of Human Morphology and Embryology, Wroclaw Medical University, T. Chałubińskiego 6a, 50-368 Wrocław, Poland; 3 Nanores Sp. Z O. O. Sp. K, Bierutowska 57-59, 51-317 Wrocław, Poland; 4 Institute of Combustion Engines and Powertrains, Poznan University of Technology, Pl. Marii Skłodowskiej-Curie 5, 60-965 Poznań, Poland

**Keywords:** ESEM, Cell morphology, Gas chromatography, Internal combustion engine, Emission, Mechanical engineering, Ecology

## Abstract

Exhaust emissions, which count among the most common causes of premature death worldwide, can cause irreversible changes in cells, leading to their damage or degeneration. In this research, L929 line cells were observed after exposure in the BAT-CELL chamber to exhaust gases emitted from a Euro 6 compression-ignition engine. Real road traffic conditions were simulated, taking into account air resistance while driving at speeds of 50 km/h, 120 km/h and idling engine. Morphological analysis of the cells was performed using an environmental scanning electron microscope. It has been observed that diesel exhaust fumes can cause inflammation, which can induce apoptosis or leads to necrotic cell death. The impact of the vehicle exhaust gases can inhibit cell proliferation by almost three times. Moreover, a correlation has been observed between the speed of the inflammatory reaction in cells and the presence of specific hydrocarbon compounds that determine the toxicity of exhaust gases. Research has shown that the toxicity of the emitted exhaust gases has been the highest at the driving speed of 120 km/h. In order to reduce the harmful effects of exhaust emissions, ecological alternatives and the supplementation of legal provisions regarding the compounds subject to limitation are necessary.

## Introduction

In 2022, 10 years have passed since the World Health Organization officially recognized exhaust gases emitted from vehicles equipped with compression-ignition engines as carcinogenic. However, they still represent a significant 37% share among all vehicles registered in Poland^1^. Despite the continuous electrification and hydrogenation of transport ^2,3^, utilization of combustion engines as an energy source is expected to remain a topical issue for many years to come, especially in the context of evaluation of emitted gases and their impact on living organisms. Results of the current research indicate that various exhaust gases, despite meeting emission regulations, have significan^4^ and varied^5^ toxic effects. Numerous times it has been scientifically proven that the toxicity of engine exhaust gases is determined by hydrocarbons^6^. Among them, it is possible to distinguish from several dozen to several hundred different compounds which, when present in relatively small amounts, often cause carcinogenic, mutagenic or genotoxic reactions^7^, and their toxicity may be additionally intensified by the phenomenon of additive synergism^8^.

The current state of knowledge does not provide sufficient information about the cellular mechanisms that occur during exposure to various types of air pollutants^9^, including gases emitted from combustion engines^10^. Current in vitro studies on the impact of exhaust gases on living cells do not usually consider the immediate effects in the cells' reaction to toxins^11,12^. The only known effects are the cytotoxic and genotoxic effects caused by acute exposure of A549 cells to gasoline engine exhaust, which include cell membrane damage, DNA damage leading to the induction of apoptosis and reduced cell viability^10^. Low vehicle operating temperatures were also found to significantly increase emissions^13^ and have negative effects on A549 and THP-1 cell lines^14^. Emission analyzes are often based on tests of vehicles with different after-treatment systems and using different fuels. Scientists used ultrafine particles with a diameter of ≤ 0.1 μm collected from exhausts of a heavy-duty-engine run with renewable diesel (A0) and fossil diesel (A20), and from a modern diesel vehicle run with renewable diesel (Euro 6) and compared their health effects on the primary human olfactory mucosa cells by assessing cellular processes on the functional and transcriptomic levels. Exposure to A0 and A20 induced substantial alterations in processes associated with inflammatory response, xenobiotic metabolism, olfactory signaling, and epithelial integrity. Euro6 caused only negligible changes, demonstrating the efficacy of aftertreatment devices^15^. It has also been shown that the composition of hydrocarbons impacts the changes in the number of cells occurring after exposure to a mixture of exhaust gases emitted from the tested passenger vehicles^16^. The more diverse the qualitative and quantitative composition of polycyclic aromatic hydrocarbons (PAHs) and volatile organic compounds (VOCs), the more cells degenerate. The toxicity of individual compounds in the group of hydrocarbons (limited as a sum) has been proven to differ significantly (up to a thousand times), as indicated by the maximum allowable concentration (MAC) values^17^ and toxicity coefficients^18,19^. Therefore, reducing the concentration of hydrocarbons in exhaust gases does not affect their actual toxicity, which should be interpreted as the harmful effect of a given substance on living organisms or biological processes.

The presented studies allow us to assume that the impact of certain compounds from the hydrocarbon group may cause irreversible changes in cells leading to their damage or degeneration. Therefore, engine exhaust fumes might be considered a cell death inducing factor. Cells undergoing apoptotic and necrotic death can be distinguished based on their morphological analysis. Apoptosis can be compared to the planned, controlled removal of certain cells, which in turn contributes to the development, homeostasis and well-being of the entire organism. Unlike necrosis, which is caused by an external pathological factor, apoptosis is a natural phenomenon in the development and life of organisms. Nevertheless, it has been shown that some pathogens can influence also the induction of apoptosis process^20^.

Microscopic and cytometric techniques deserve special attention when assessing the mechanism of cell death^20^. Light microscopes with relatively low magnification can provide valuable information about changes in the size of exposed cells. Electron microscopes enable the observation of external cell features characterizing death by apoptosis or necrosis^21^. It is possible to observe more typical internal cell features when transmission microscope imaging is utilized. Additional use of cytometric techniques, which provide information about the cell survival rate and the course of the cell cycle or proliferation, allows drawing more complete conclusions about the health of the cells exposed to toxic gas mixtures.

As is known, the current assessment methods for the quality of emitted exhaust gases, used when allowing vehicles to traffic, do not include the measurement of the most toxic hydrocarbons^22^. Based on the current methods, it is also not possible to draw conclusions about the harmful effect the actual toxicity of the exhaust gases has on humans^8^.

The aim of this research was to assess the morphology of L929 line cells after exposure to engine exhaust gases using scanning electron microscopy and to compare the results with the concentration values of hydrocarbons emitted in the exhaust gases. The acute toxicity—the immediate effect of the gas mixture on L929 fibroblasts was tested.

## Materials and methods

### Research object

The tests covered exhaust gases emitted from a BMW 120 d x-drive passenger vehicle equipped with a diesel engine. The technical parameters of the vehicle are presented in Table 1. The vehicle has passed a technical inspection at a vehicle inspection station. An additional inspection was carried out at the testing site in order to ensure no defects of the vehicle. The inspection included a visual assessment of the engine compartment for leaks or irregularities in the operation of power unit components and for the tightness of the exhaust system.

**Table 1:** Technical parameters of a vehicle with a diesel engine.

Fuel type	Ekodiesel ULTRA
Displacement engine	1995 cm^3^
Maximum power	140 kW at 4000 rmp
Maximum torque	400 Nm at 1750–2500 rpm
Actual vehicle weight	1470 kg
Drive type	4 × 4
Gearbox type	Automatic, 8 gears
Year of production	2017
Exhaust emission level	EURO 6b
Vehicle mileage	90,267 km

The vehicle was approved before 2017 during the period of validity of the Euro 6 standard in the version without updates. The vehicle was powered by commercially available Ekodiesel ULTRA diesel oil with a biocomponent content not exceeding 7%.

### Method of exhaust gas sampling

The collection of exhaust gases emitted from the vehicle was carried out on the MAHA MSR 1050 chassis dynamometer. The test station, due to its ability to generate braking and driving torque, allows for simulating the vehicle's movement resistance. In order to determine the vehicle's movement resistance (rolling and air resistance coefficients), a coastdown test was performed. The vehicle's inertial resistance was determined by measuring the actual vehicle mass. The inertia component in this case was negligible, measurements were carried out for a constant vehicle speed. Data from the tests was implemented into the test station's software, which, through the appropriate distribution of torque, simulated the vehicle's motion resistance during operation in real conditions. Before the main measurements have been performed, the external characteristics of the vehicle were determined, and the emptying of the particulate filter was triggered so that the filter cleaning procedure would not occur during the actual tests.

The tests were carried out in three vehicle states: while stationary (engine idling), while driving at a speed of 50 km/h, and at 120 km/h (drive system ratio according to the gearbox control algorithm).

Each time, the engine and drive system were running at a temperature typical for a given condition. The conditions were met by maintaining the vehicle at a defined operating point for a period of 15 min before the start of the tests until the vehicle's thermal equilibrium was achieved.

To collect exhaust gases, a silicone pipe with a metal extension was placed in the vehicle's exhaust pipe. The other end was connected to an inert sampling bag placed in a special aspiration system.

For each vehicle condition, exhaust gases were collected into six prepared bags with a capacity of 10 dm^3^ in order to examine VOCs (1 bag), PAHs (3 bags) and exhaust gas toxicity in in vitro tests (2 bags). During sampling, the emission level of harmful exhaust gas components was monitored using an automatic exhaust gas analyzer MAHA MET 6.1, equipped with an NDIR infrared sensor to monitor the concentration of carbon monoxide, carbon dioxide and hydrocarbons and an electrochemical oxygen sensor.

### Cell culture

The research used the adherent fibroblast-like cell line L929 (ECACC 85,011,425) obtained from the subcutaneous adipose tissue of mice, which can be used for toxicity testing. This line is a reference model used in the assessment of the cytotoxicity of biomaterials in accordance with the ISO-10993:5 standard^23^.

#### Carrying out a cell culture

Cell culture was carried out using standard operating procedures^24^. The culture was carried out in MEM (Minimum Essential Medium) with Earle's Salts (Capricorn Scientific, Ebsdorfergrund, German) enriched with 10% fetal bovine serum (FBS, Sigma-Aldrich, St. Louis, MO, USA) and 1% L-glutamines, penicillin, streptomycin and an acidity regulator HEPES (Sigma-Aldrich). The culture was carried out in a humid atmosphere under standard conditions: 37 °C, 5% CO_2_ in a CELL 50 Comfort S CO_2_ incubator. Cells were passaged using 0.25% trypsin–EDTA solutions (Sigma-Aldrich) at 70% confluence. Control imaging of cells during culture was performed using an AE31E trino inverted microscope with a maximum magnification of 400 times, equipped with a Moticam Pro S5 Lite camera.

#### Exposure of cells to exhaust gases

24 h before exposure to exhaust gases, cells were seeded into bottles with adherent medium with an area of 25 cm^2^ at a density of 23,000 cells/cm^2^. Cells were quantified using an EveTM NanoEn Tek Inc. automatic cell counter.

The BAT-CELL Bio-Ambient-Tests method (patent, PL, No. 220670) was used to expose cells to a mixture of exhaust gases. The method enables the assessment of the toxic effect of gas mixtures on the health of living organisms. The prepared cell line, devoid of culture fluid, is placed in a sterile sampler. Then, the aspiration system draws exhaust gases into the sampler through an inlet pipe equipped with an antibacterial filter that acts as a barrier to particulate matter. After exposure, the cells are flooded with culture fluid and the direct effect of toxic gases on living cells is examined using toxicological tests, for instance viability test. The exposure time is selected individually depending on the type of gas mixture being tested. For exhaust gases, the exposure time was set to 7.5 min^16^. The flow parameters are adjusted to the shape of the sampler in such a way as to enable uniform contact of the gas particles with the cell surface and not to damage them mechanically. The flow rate of tested gases through the aspiration system was set at 150 cm^3^/min^25^.

The conditioning chamber in which the samplers were placed was equipped with pressure and temperature sensors to ensure that the vital functions of the cell culture had been maintained. Elimination of the culture fluid was possible due to maintaining physical parameters, adjusted to the requirements of a given cell line, safe for cells outside the incubator atmosphere for a specified period, and without the supply of nutrients. The cell line sampler was additionally protected at the inlet with an antibacterial filter. Three samples were exposed to each of the prepared simulated vehicle conditions and two control tests were carried out: a sampler with cells left in the laboratory and a sampler with cells exposed to clean air.

The repeatability of the BAT-CELL Bio-Ambient-Tests method was estimated at the level of 5%, which is a relatively low value compared to other biological methods^26^. The method error is related to the average reading error value from the tested samples and was determined during previous experiments.

#### Determination of cell viability

Cells were counted 48 h after exposure to the tested gas mixture^16^ using trypan blue (Sigma-Aldrich) and an automatic cell counter. The total number of cells, the number of living cells, and the number of dead cells were counted. Cell survival was presented as the percentage of viable cells relative to all cells in the airflow control. The color change of the culture fluid immediately after exposure to toxins was also assessed.

### ESEM imaging

The morphology of L929 cells was imaged using a Quanta 3D 200i ESEM/Ga-FIB microscope, which combines an electron microscope based on a tungsten cathode and an ion microscope. The microscope used has three imaging modes, including low vacuum (Lo-Vac), which was used in the research. A Large Field Detector (LFD), which collects signals from both secondary and backscattered electrons, was used. Photographs of the cells were taken 24 h and 48 h after exposure to the exhaust gases for each vehicle condition and for the control sample (cells not exposed to exhaust gases). All samples were viewed at 3000 × magnification and a pressure of 70 Pa. Detailed imaging parameters are provided under each photograph in the following chapter.

Before imaging, cells stuck to the adherent substrate were fixed with methanol and stored for 24 h in Dulbecco's PBS solution (Capricorn Scientific, Ebsdorfergrund, German) at 4 °C until the imaging time. Before placing the preparation on the microscope stage, the adherent surface was rinsed with distilled water to wash away the crystallized PBS solution.

### Gas chromatography

Qualitative and quantitative analysis of the groups of volatile organic compounds and the polycyclic aromatic hydrocarbons was performed using gas chromatography Samples for the analysis of VOCs and PAHs were collected with a two-channel automatic aspirator ASP II from LAT using an SKC LOT 120 sorbent with a gas flow rate of 30 dm^3^/h. Samples with activated carbon were stored at a temperature below 20 °C until they were submitted to laboratory tests for the presence of VOCs and PAHs. Carbon disulfide was used as the extracting substance.

A VARIAN 450GC gas chromatograph with a flame-ionization detector and a capillary column (Varian VF-WAXms 30 m × 0.25 mm ID DF: 0.25 μm) was used for the quantitative and qualitative analysis of the VOCs. The work was carried out at the temperature of the column fixed at 373 K (110 °C), the temperature of the injector fixed at 423 K (150 °C), and the temperature of the detectors fixed at 423 K (150 °C).

PAHs were determined using a mass detector (MS). This method utilizes the SPE solid phase extraction technique. A C18 chromatographic column with the smallest possible grain sizes and porosity was used. In the case of PAHs, dichloromethane was used as the extracting substance. A C18 chromatographic column with the smallest possible grain sizes and porosity was used. In the case of PAHs, dichloromethane was used as the extracting substance. Furnace temperature program: 45 °C (0.8 min), temperature increase from 45 °C/min to 200 °C, temperature increase from 2.55 °C/min to 225 °C, temperature increase from 3 °C/min to 266 °C, then temperature increase from 5 °C/min to 300 °C (5,289 min). The analysis time lasted 40 min.

Before the analysis of VOCs and PAHs, the devices were calibrated, and the recovery of the methods was established by determining the calibration coefficients and desorption coefficients. The total relative error of the VOC analysis method was estimated at 20%^27^, and for the PAHs at 30%^28^.

## Results

### Chemical analysis of hydrocarbons contained in exhaust gases

Concentrations of individual compounds present in the exhaust gas mixture were calculated based on the data read from the chromatograms. 5 compounds from the PAH group and 4 from the VOC group were detected in the exhaust gases of a vehicle traveling at a speed of 120 km/h.

The list of values for the highest permissible concentrations of chemical and dust factors harmful to health in the work environment includes the sum of the concentrations and carcinogenicity factors of nine carcinogenic PAHs at the MAC level = 0.002 mg/m^3^^17^. In the tested vehicle equipped with a diesel engine, five of them were detected, including dibenzo(a,h)anthracene with the highest carcinogenicity factor and ane(a)pyrene, which is a representative of the PAH group (Table 2). The MAC value for the identified compounds was 15,702 mg/m^3^, which exceeded the permissible MAC value by several thousand times.

**Table 2:** Concentration of PAHs in the exhaust gases of a vehicle moving at a speed of 120 km/h.

z	Concentration in the exhaust [mg/m^3^]	Carcinogenicity factor value *k*^17^	Concentration * *k* [mg/m^3^]
Chrysene	0.573	0.01	0.006
Benzo(a)pyrene	1.789	1	1.789
Indeno(1,2,3-cd)pyrene	2.119	0.1	0.212
Dibenzo(a,h)antracene	2.736	5	13.680
Benzo(ghi)perylene	1.455	0.01	0.015
Sum:			15.702

Additionally, by comparing the obtained value of benzo(a)pyrene concentration with the average annual permissible concentration in atmospheric air established in the EU, which is 1 ng/m^3^^29^, it can be assumed that in a crowded urban agglomeration, the measured value of this compound exceeds the permissible standards.

It is important to prepare comparisons of such sort, as people in modern societies are constantly exposed to emissions of toxic compounds from engine exhaust gases. The best example of a group at risk is professional drivers who spend almost half of the day in the vehicle cabin, including about 1.5 h a day in road congestion conditions^30^. Residents and employees of large cities, especially people who often stay in places with heavy traffic, are also exposed to the effects of the long-term impact of harmful exhaust gases. Hydrocarbons are present not only in car engine exhaust gases but also in gasoline, tobacco smoke, food products, and drinking water, the degree of exposure increases, becoming a significant social problem^31^. It should be emphasized that the absorption of PAHs through the skin may constitute a significant risk, as in the case of inhalation exposure^17^.

Table 3 lists the volatile organic compounds detected in exhaust gases. The NDS values for the VOCs were not exceeded. For example, for ethyl acetate, the limit is NDS = 734 mg/m^3^. In addition to ethyl acetate and isobutanol, trace amounts of hexane and benzene were recorded. Due to the concentration values of these compounds being below the chromatograph calibration curve, their exact value has not been determined. In high concentrations, the detected compounds may cause irritation to the skin, eyes, and respiratory system, and cause dizziness, drowsiness, nausea, or even arrhythmia. Compounds are present only in trace amounts does not necessarily mean that they have no harmful effects on the body. They can form multi-component mixtures, and such configurations may enhance their toxic effect.

**Table 3:** Concentration of VOCs in the exhaust gases of a vehicle moving at a speed of 120 km/h.

Compound detected	Concentration in the exhaust [mg/m^3^]
Ethyl acetate	691.2
Isobutanol	563.4
Hexane	+ *
Benzene	+ *

*concentration below the measuring range.

The concentrations of carcinogenic PAH compounds significantly exceeded the values specified by the standards. The driver in the vehicle cabin is exposed to much smaller amounts of toxic compounds compared to the amount emitted directly from the vehicle's exhaust pipe. However, it should be noted that in addition to the emissions from exhaust gases, in the vehicle cabin, the driver and passengers are exposed to emissions from other sources, for example from vehicle interior trim elements made of plastic. Vehicle emissions also include abrasion of brake pads, tires, and clutch, losses during refueling, and suspension wear. Total concentrations of harmful compounds emitted from vehicles powered by fossil fuels constitute the largest source of transport pollution^32^.

### Engine exhaust cytotoxicity

**Figure 1:**
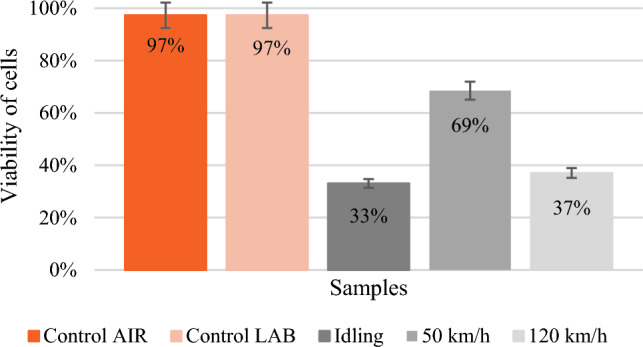
Cells viability 48 h after exposure.

Table 4 shows the information obtained from the automatic cell counter. Figure 1 presents the cell survival 48 h after exposure to engine exhaust gases for three different vehicle conditions and in the control tests. Control AIR means a sampler through which clean air was passed, and control LAB means a sampler that was left in the incubator. Survival for the control trials was at 97%, which is the optimal, desired result. The highest survival rate of 69% was recorded for the test in which the vehicle moved at a constant speed of 50 km/h. Approximately twice lower results were obtained for the idle run test and the speed of 120 km/k, 33% and 37% respectively.

**Table 4:** Information from the automatic cell counter.

Samples	Total [× 10^6^]	Live [× 10^6^]	Dead [× 10^5^]	Viability ()
Control AIR	3.6	3.5	1.2	97
Control LAB	3.8	3.5	1.1	97
Idling	1.3	1.1	1.0	31
	1.6	1.5	0.9	42
	1.1	1.0	0.8	27
50 km/h	2.8	2.7	1.0	75
	2.9	2.8	1.1	78
	2.0	1.9	1.2	53
120 km/h	1.3	1.2	1.3	33
	1.5	1.4	1.3	39
	1.7	1.4	1.7	39

**Figure 2:**
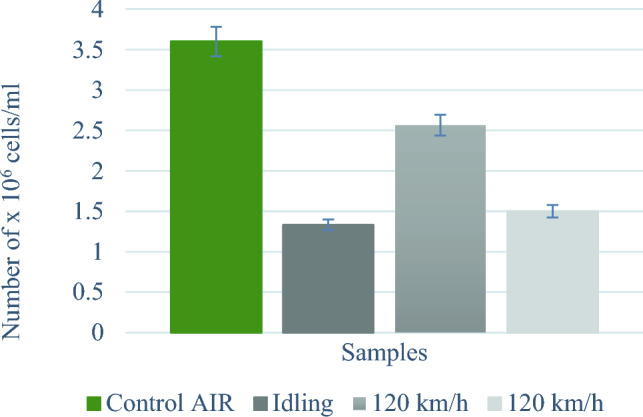
Total number of cells attached to the growth surface 48 h after exposure.

Additionally, the total number of cells attached to the growth surface, remaining in the samplers after exposure and thus capable of proliferation was counted. In the figure (Fig. 2) the average total number of cells for individual vehicle states. Clear disparities had been noticed. 48 h after exposure to exhaust gases, there were 3.6 × 10^6^ cells/ml in the control sample. For idling, the average observed value was 1.3 × 10^6^ cells/ml, for a vehicle moving at a speed of 50 km/h – 2.6 × 10^6^ cells/ml, and for a vehicle moving at a speed of 120 km/h – 1.5 × 10^6^ cells /ml. Lower cell adhesion to the growth surface in the samplers subject to exposure to exhaust gases may indicate that the exposure inhibits the cells' ability to reproduce. At idle speed, the number of cells decreased almost three times, at 50 km/h by almost half, and at 120 km/h by more than twice in relation to the number of cells in the control sample. The error bars in Figs. 1 and 2 refer to the 5% method error value mentioned in Section "Cell culture".

**Figure 3:**
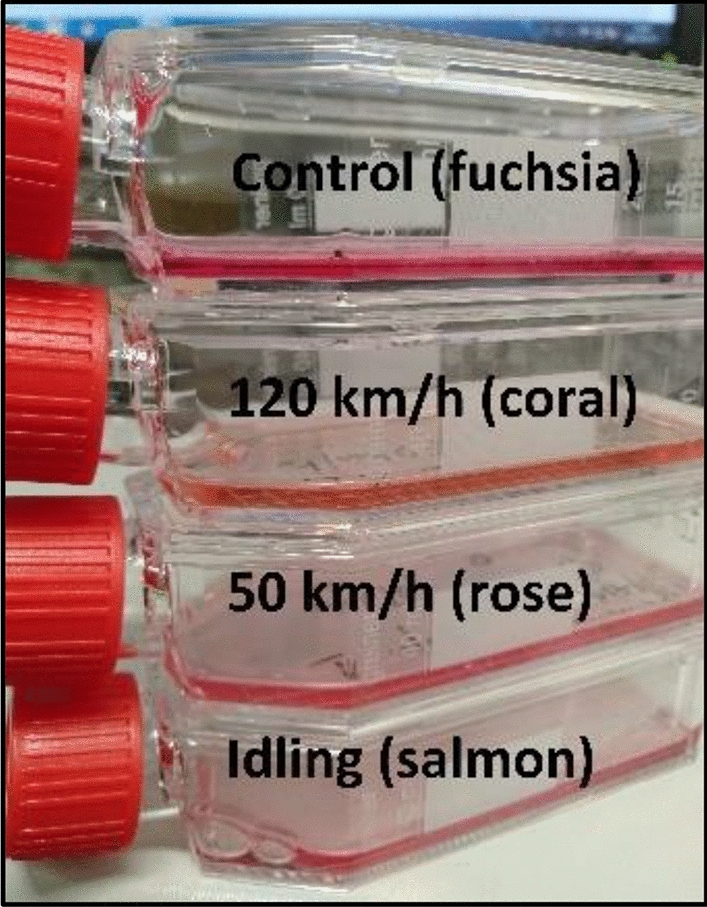
Change in color of the culture fluid immediately after exposure of the cells to exhaust fumes.

Changing the pH of the cell environment has a significant impact on the cell health. Immediately after exposure to exhaust gases, the culture fluid poured into the cells changed color significantly compared to the control sample with the clean air flow (Fig. 3). The reaction of condensate formed from the water vapor from exhaust gases is acidic. The sampler's microenvironment was probably acidified, which caused a change in the color of the solution. In future tests, it should be considered to measure the pH of fluids with a pH meter.

### Imaging of cells

**Figure 4:**
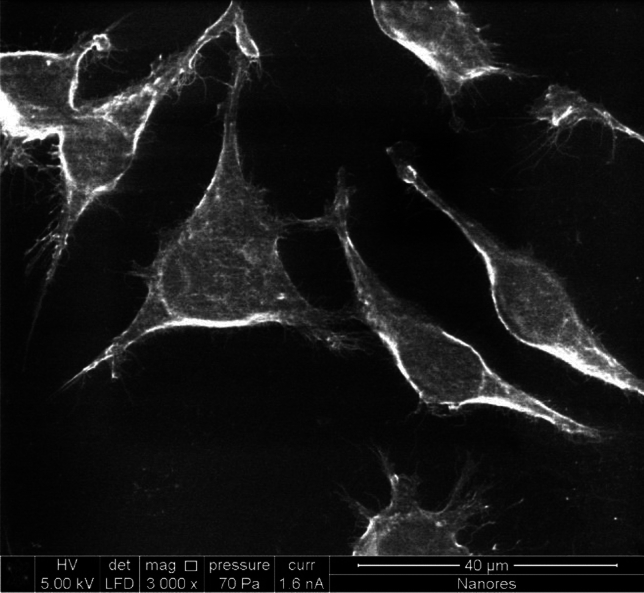
Control sample – healthy cells not exposed to exhaust fumes.

Figure 4 shows cells that were not exposed to exhaust gases. The image was taken using environmental scanning electron microscope (ESEM) in low-vacuum imaging mode. The image shows inactivated fibroblasts with visible collagen fibers. The cells grew as a normal two-dimensional monolayer, revealing a spindle-shaped morphology. The image also shows cell mitosis (division in late telophase) and daughter cells connected by a remnant body. The cell length can be estimated at approximately 40 µm.

**Figure 5:**
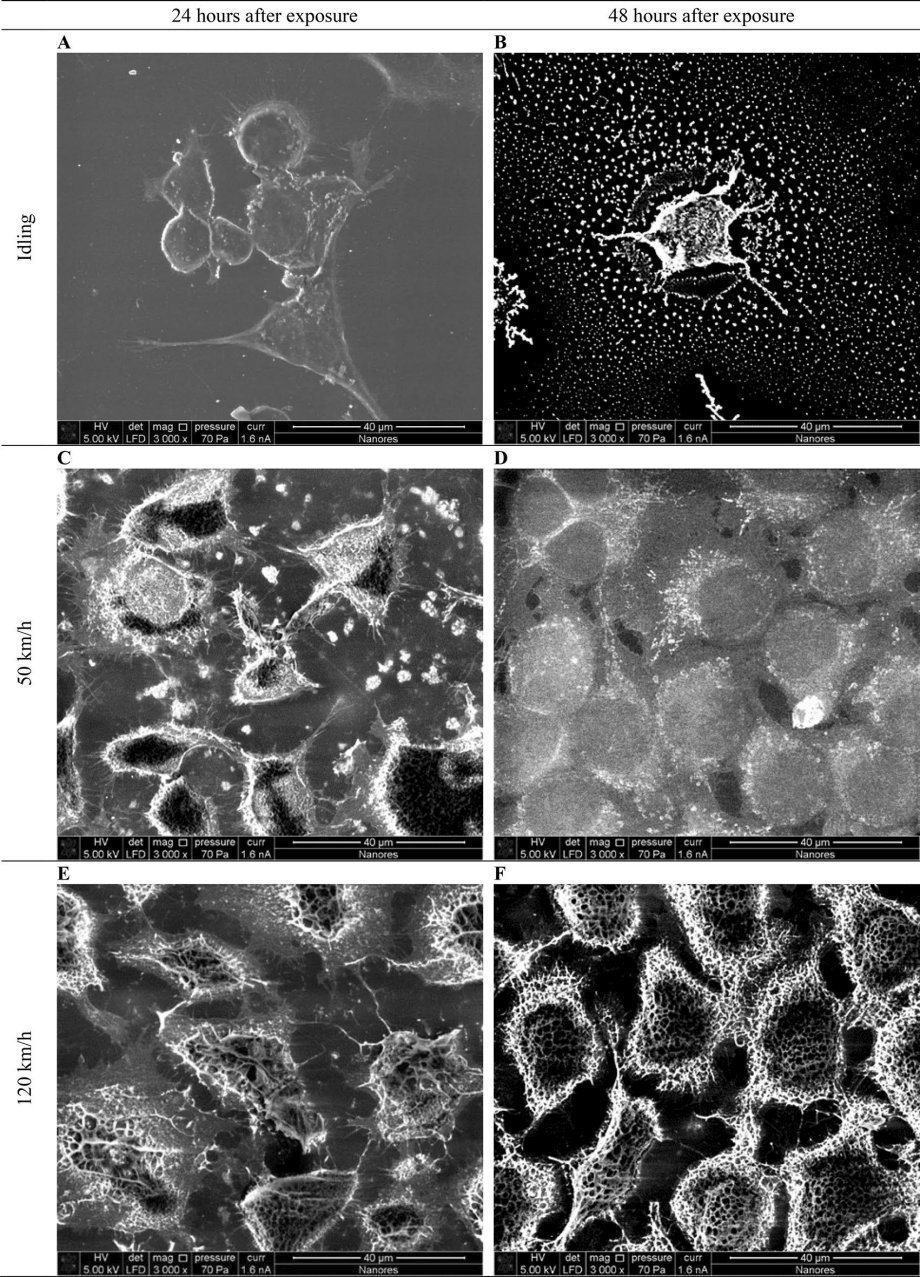
Cell morphology after exposure to diesel exhaust.

Figure 5 shows the morphology of cells 24 h and 48 h after exposure to exhaust gases using ESEM. Based on the literature, published photographs, and results of similar research^20,24^, it was established that the cells exposed to engine exhaust fumes show morphological features of apoptotic or necrotic cells. Photograph A presents the cells 24 h after exposure to exhaust fumes coming from an idling vehicle. Spherical cells in the final phase of mitotic division and apoptotic bodies forming are visible in the photograph. Photograph B presents a spherical cell with many apoptotic bodies around the cell. The formation of apoptotic bodies is typical for cells dying by apoptosis. In addition, shrinkage of collagen fibers can be observed. Photograph C presents the cells 24 h after exposure to exhaust fumes from a vehicle traveling at a constant speed of 50 km/h. Darker areas of the cytoplasm can be observed as a result of the cell losing water, thus causing the cell's cytoplasm to thicken. Transitional microvilli are visible on the cell membrane in the form of finger-like projections of various lengths, the length and density of which may change depending on the functional state of the cell or the phase of the mitotic division^20^. Apoptotic bodies are also present. Photograph D presents the cells 48 h after exposure to exhaust fumes from a vehicle moving at a constant speed of 50 km/h. Traditional electron microscopy allows the imaging of well-conducting samples. The use of the low vacuum mode enabled the imaging of a sample with low conductivity but did not allow for obtaining the resolution expected for well-conducting samples. In photograph D it is possible to observe an increase in the volume of cells that have taken on a spherical shape, which is typical for cells dying because of necrosis. The necrotic cell swells as a result a of partial breakdown of the cell membrane and ingress of water into the cytoplasm, which is the opposite of the apoptotic process^20^. Differences in the color of the cytoplasm are visible—lighter swollen cells and slightly darker cells with visible breakdown of the cell membrane. As a result, cytoplasmic components flow into the intercellular environment. Photographs E and F present the cells with structural disintegration of the cell membrane. These are cells that were exposed to exhaust fumes from a vehicle moving at a constant speed of 120 km/h. Collagen fibers have degenerated, cell volume has increased compared to cells not exposed to exhaust fumes, and transitional microvilli are present. The shape of the fibroblasts indicates inflammation, leading to necrosis.

**Figure 6:**
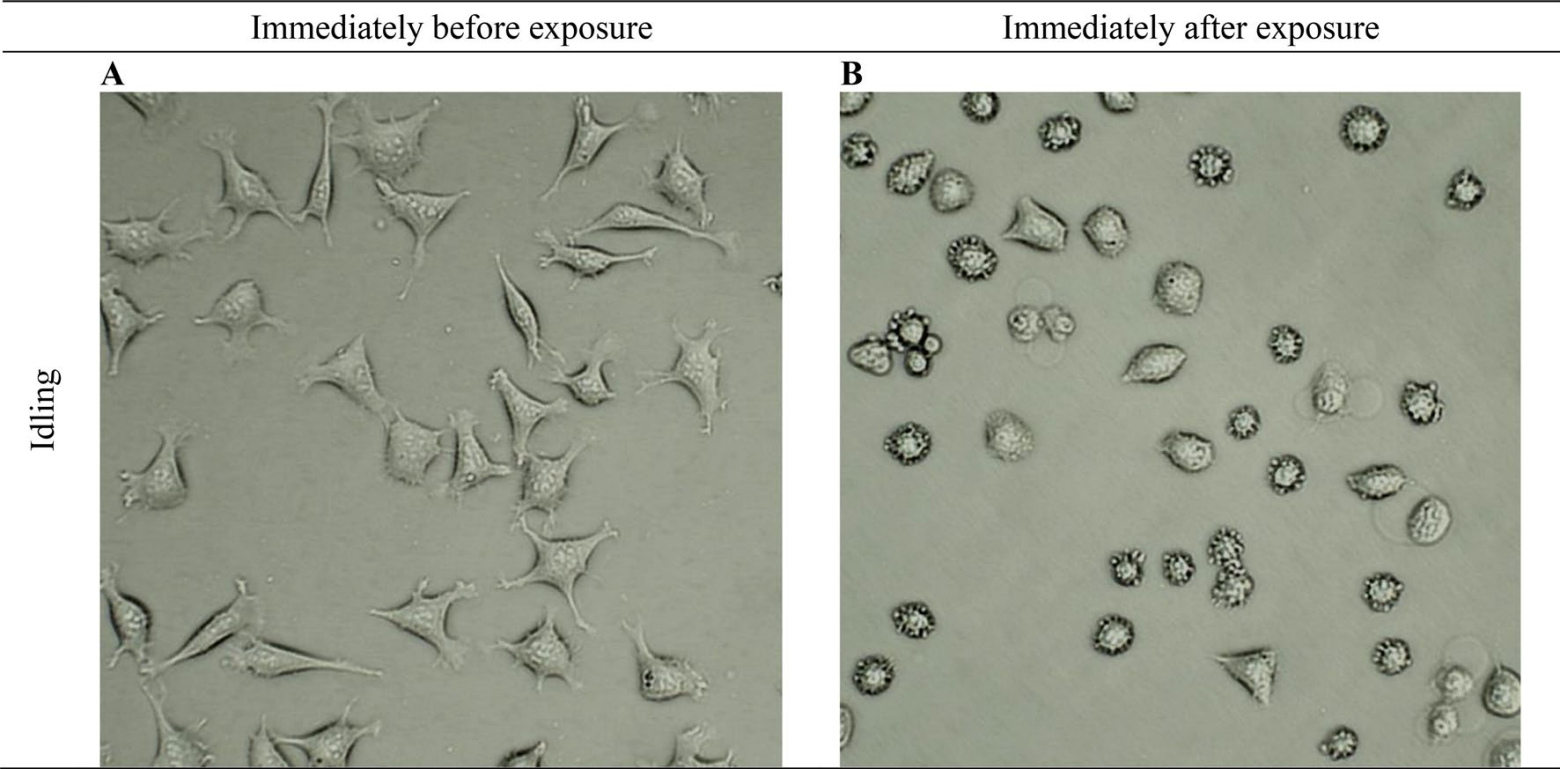
Cell morphology immediately before and after exposure to idling diesel exhaust; × 40 zoom.

Figure 6 presents photographs taken with an optical microscope with 200 × magnification. Cells were imaged immediately before and immediately after exposure to the exhaust fumes coming from an idling vehicle. Immediately after a 7.5-min exposure to exhaust gases, cell shrinkage and the formation of apoptotic bodies were observed in most cells. It is therefore probable that engine exhaust gases emitted at idle speed induce the process of apoptosis in cells.

**Figure 7:**
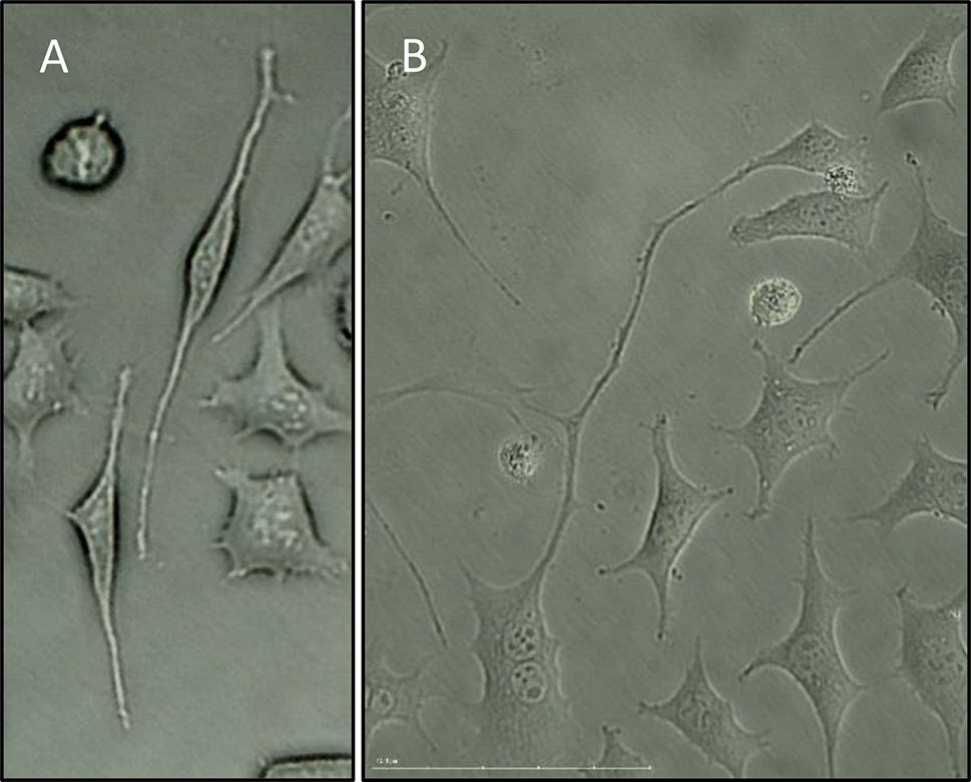
Stretching of collagen fibers: (A)—immediately after exposure to diesel exhaust fumes traveling at a constant speed of 120 km/h; (B) – 72 h after exposure to idling diesel exhaust; × 40 zoom.

Figure 7 presents the cells with unusual stretching of collagen fibers because of exposure to exhaust gases. This effect was observed in many cells immediately after exposure to exhaust fumes from a vehicle moving at a speed of 120 km/h and 72 h after exposure to exhaust fumes from an idling vehicle.

## Discussion

The conducted research indicates the harmful impact of engine exhaust gases emitted from the tested vehicle equipped with a diesel engine and meeting the current Euro 6 exhaust emission standard on the L929 line cells. To draw full conclusions about the mechanisms of cell death through apoptosis or necrosis after exposure to engine exhaust fumes, additional tests should be performed in future studies, including Annexin/PI flow cytometry or the more accurate TEM imaging. Simultaneous studies of the cell cycle and apoptosis in cells should be performed and included in the integral analysis^33^. Nevertheless, based on the presented preliminary results, the following conclusions were formulated:

1. Cells of the L929 line exposed to the tested exhaust gases may show morphological characteristics of apoptotic and necrotic cells, which is associated with their survival and ability to proliferate being reduced up to several times.
2. Engine idling may induce the apoptosis process in L929 cells immediately after exposure to the tested exhaust gas mixture, and the duration of the changes in cells is not transient.
3. The tested engine exhaust gases emitted while driving at a constant speed of 120 km/h may lead to necrosis of L929 cells, which may be related to the presence of highly carcinogenic PAHs in the exhaust gas mixture.

The tested vehicle meets the applicable European exhaust emission standards. However, these standards do not directly take into account the compounds, which are not subject to limitation but determine the toxicity of engine exhaust gases. These compounds are hydrocarbons, the concentration of which is determined only for the sum of the entire group, and there are no tests of their qualitative composition. Among particularly harmful hydrocarbons, two groups of compounds deserve attention: polycyclic aromatic hydrocarbons (PAHs)^34^ and volatile organic compounds (VOCs)^35^, because many substances belonging to these groups have proven highly toxic, carcinogenic, mutagenic or genotoxic effects. Nitrogen oxides (mainly nitrogen oxide NO and nitrogen dioxide NO_2_)^36^ and carbon monoxide CO^37^ are single compounds directly covered by emission limits and well recognized in terms of toxicology. In the future, it is important to determine the significance of the impact of all individual gas components on the actual toxicity of exhaust gases. It is possible that the low cell survival rate for idling and at 120 km/h is a result of the negative interaction of various substances in the exhaust gas. An additional increase in the concentration of VOCs and PAHs at 120 km/h may be the result of triggering DPF regeneration, but these are only speculations for which the authors do not have confirmation in the test results. Therefore, further research in this direction is advisable.

The European exhaust emission standards also do not take into account the actual impact of exhaust gases on living organisms. The currently used emission control methods for admitting vehicles to traffic, as well as inspection methods at vehicle inspection stations, seem to not be adequate and do not solve the existing problem. It would be advisable to supplement the current methods with more detailed analyses of the composition of the exhaust gas mixtures, especially the hydrocarbon content. It is also necessary to consider the use of alternative methods, such as testing the actual toxicity of exhaust gases, in order to determine their actual harmful impact on the human body. The use of gas chromatography and biological methods based on in vitro tests may be an appropriate complement to the exhaust emission control methodology.

## Data Availability

The datasets generated during and analyzed during the current study are available from the corresponding author on reasonable request.
